# Mogamulizumab Suppresses Induction of Myelin-Reactive Antibodies After Experimental Spinal Cord Injury

**DOI:** 10.3390/cells15211999

**Published:** 2026-10-31

**Authors:** Darya A. Orlova, Aleksandr S. Chernov, Vitaly A. Kazakov, Georgii B. Telegin, Alexey A. Belogurov, Anna A. Kudriaeva

**Affiliations:** 1 Shemyakin and Ovchinnikov Institute of Bioorganic Chemistry, Russian Academy of Sciences, Moscow 117997, Russia; dorlova01@yandex.ru (D.A.O.); alexandrchernov1984@gmail.com (A.S.C.); 2 Moscow Center for Advanced Studies, Kulakova Str. 20, Moscow 123592, Russia; 3 Branch of Shemyakin and Ovchinnikov Institute of Bioorganic Chemistry, Russian Academy of Sciences, Pushchino 142290, Russia; vitalij.tomsk@list.ru (V.A.K.); telegin@bibch.ru (G.B.T.); 4 Federal Budget Institution of Science «State Research Center for Applied Microbiology and Biotechnology» a Part of the Federal Service for Supervision of Consumer Rights Protection and Human Welfare of the Russian Federation (FBIS SRCAMB), Obolensk 142279, Russia; 5 Department of Biological Chemistry, Russian University of Medicine, Ministry of Health of the Russian Federation, Moscow 127473, Russia

**Keywords:** spinal cord injury, cytokines, chemokines, immunoproteasome, treatment, mogamulizumab

## Abstract

**Highlights:**

**What are the main findings?**

- β1i-enriched immunoproteasome and PA28β regulatory particles are induced in the injured spinal cord within 12 h of SCI and track IL-1α, not IFN-γ.
- Mogamulizumab lowers anti-MBP antibody levels and reduces glial scar area and cystic cavity formation after SCI.

**What are the implications of the main findings?**

- Early immunoproteasome induction temporally precedes the myelin-reactive humoral response after SCI and may contribute to its development
- Anti-CCR4 therapy offers a complementary immunological strategy for spinal cord protection after injury.

**Abstract:**

Traumatic spinal cord injury (SCI) is believed to increase the risk of autoimmune diseases, including the autoimmune demyelinating disease multiple sclerosis (MS), in humans. The adaptive immune response against one of the key MS autoantigens, myelin basic protein (MBP), is elevated in mice and persists in patients with SCI even 10 years after the initial injury. Here, we show that β1i-enriched immunoproteasomes and PA28β subunits are elevated as early as 12 h post-SCI in DA rats, and all proteasome catalytic immunosubunits are highly abundant in the injured spinal cord of DA and SD rats 7–14 days post-SCI; these changes may therefore represent a primary event of the adaptive myelin-reactive immune response. Mogamulizumab bound a recombinant rat CCR4 N-terminal extracellular fragment, albeit less strongly than the corresponding human fragment, and treatment was associated with weaker CCR4-associated immunoreactivity in peripheral blood leukocyte lysates. Plasma samples from rats with SCI treated with mogamulizumab were characterized by lower levels of antibodies to MBP compared with those from the non-treated group, and by day 60 of the experiment, this discrepancy reached statistical significance. Compared with non-treated animals, rats treated with mogamulizumab showed a significantly decreased glial scar size and total area of cyst-like cavities. Thus, mogamulizumab therapy not only improves the morphological features of the glial scar but also suppresses the autoimmune response against myelin, and may offer a complementary immunological strategy for spinal cord protection following injury.

## 1. Introduction

Spinal cord injury (SCI) is a severe neurological condition characterized by complex pathophysiological changes leading to severe physical impairment [1]. The pathogenesis of SCI consists of two linked processes: primary mechanical damage caused by the immediate traumatic impact, and secondary damage developing over the subsequent hours and days [2]. Cellular injury and blood–spinal cord barrier (BSCB) disruption trigger an inflammatory cascade, which causes neuroinflammation, ischemia, excitotoxicity, oxidative stress, and neuronal apoptosis, and eventually leads to glial scar formation and permanent loss of neurological function [3].

The cytokine storm that occurs during trauma plays a crucial role in the pathogenesis of secondary injury. Recently, we showed that SCI in mammals is mainly driven by a massive cytokine storm in the cerebrospinal fluid (CSF) [4]. The acute phase was characterized by the release of CXCL1/GROα, IL-6, and CCL2–5. The sub-acute phase was mostly associated with IL-2, IL-7, CCL22, and CX3CL1, whereas TNF and IL-17A permanently persisted in the CNS even weeks following SCI. These inflammatory mediators activate resident CNS cells (microglia and astrocytes), promote peripheral immune cell infiltration (neutrophils, macrophages, T cells), increase blood–spinal cord barrier permeability, and promote myelin phagocytosis [5,6]. Multiple studies report the presence of myelin-reactive T cells and, in some settings, humoral B cell responses after SCI, and such autoreactivity is thought to contribute to oligodendrocyte loss, inhibition of remyelination, and persistence of inflammation [7,8,9,10,11]. Here, we propose that therapy with the anti-CCR4 monoclonal antibody mogamulizumab could limit autoreactive responses against myelin components such as myelin basic protein.

## 2. Materials and Methods

### 2.1. Experimental Spinal Cord Injury Model

Male SD and DA rats were used to create the spinal cord injury model. The rats underwent unilateral laminectomy of the Th13 vertebra, after which the spinal cord was exposed to a cryoprobe through the dura mater of the spinal cord. The cryoprobe exposure time on the spinal cord was 1 min, and the temperature in the contact area reached −20 °C. Intact animals were used as a control group to compare the results and evaluate the effect of cryoinjury. All animals were housed under standard conditions in the Animal Breeding Facility of BIBCh, RAS (supported by the Scientific Infrastructure Project contract #075-15-2025-514). All manipulations with the animals were approved by the IACUC of the BIBCh RAS (protocol no. 913/23 of 24.01.23).

### 2.2. Spinal Cord Extract (SCE) Isolation

The animals were euthanized at different time points after the cryoinjury: 8, 12, 24 h (acute period) and 3, 5, 7, 14 days (sub-acute period). The spinal cord samples [Th12-L1 (injured site) and Th9-Th11 (adjacent area)] from the animals were homogenized in lysis buffer—PBS, 0.1% Tween-20 (PBS-T). The resulting suspension was centrifuged at 4 °C and 21,000× *g* for 30 min to separate cellular debris and obtain the supernatant, which was subjected to Western blotting. Protein concentration in the samples was determined using the Pierce BCA protein assay kit (Thermo Scientific, Waltham, MA, USA) according to the manufacturer’s protocols. All tissue manipulations were performed at 4 °C, and the SCE samples were subsequently stored at −80 °C.

### 2.3. Immunoblotting

Following SDS-PAGE, the unstained gel was transferred to a nitrocellulose membrane (Bio-Rad, Hercules, CA, USA) and then transferred by semi-dry method using a Trans-Blot Turbo (Bio-Rad, Hercules, CA, USA). After the transfer was complete, the membrane was placed in a blocking solution (5% BSA, PBS-T) and incubated for 1 h at room temperature. The membranes were then washed with PBS-T and placed in a dilution buffer (0.5% BSA, PBS-T) containing primary antibodies to PSMA2/α2 (#PA5-52527, Thermo Fisher Scientific, Waltham, MA, USA), PSMB8/β5i (#PA1-972, Thermo Fisher Scientific, Waltham, MA, USA), PSME2/PA28β (ab183727, Abcam, Waltham, MA, USA), PSMB10/β2i (EPR14902, Abcam) and PSMB9/β1i (ab3328, Abcam). After 1 h, the membrane was washed with PBS-T and then placed in a dilution buffer with secondary HRP-conjugated goat anti-rabbit antibodies (31460, Thermo Fisher Scientific, Waltham, MA, USA). The membrane was then washed with PBS-T, and blots were visualized with Clarity Western ECL chemiluminescent substrate.

### 2.4. Multiplex Assay

Multiplex assays of Th12-L1 (injured site) and Th9-Th11 (adjacent area) were performed using the Bio-Plex Pro Rat Cytokine 23-Plex assay #12005641 (Bio-Rad, Hercules, CA, USA). This assay measures 23 cytokines and chemokines: G-CSF, GM-CSF, CXCL1/GRO/KC, IFN-γ, IL-1α, IL-1β, IL-2, IL-4, IL-5, IL-6, IL-7, IL-10, IL-12 (p70), IL-13, IL-17A, IL-18, M-CSF, CCL2/MCP-1, CCL3/MIP-1α, CCL20/MIP-3α, CCL5/RANTES, TNF, and VEGF. In this panel, GRO/KC corresponds to CXCL1 and MIP-3α corresponds to CCL20; standard chemokine nomenclature is used throughout the manuscript. Sample preparation was accomplished as described previously [12]. The multiplex analysis was conducted following the manufacturer’s protocol using Bio-Plex Manager™ 6.0 software (Bio-Plex 200 System, Bio-Rad, Hercules, CA, USA).

### 2.5. Treatment of Rats with Mogamulizumab

Animals were injected intravenously with the anti-CCR4 antibody (mogamulizumab, MedChemExpress, Monmouth Junction, NJ, USA) immediately after SCI and again 5 days after injury at a dose of 4 mg/kg. Plasma samples for evaluation of anti-MBP antibodies were collected at 14, 30, 45, and 60 days after cryoinjury. Untreated rats (saline i.v. injections) with SCI were used as controls.

### 2.6. Enzyme-Linked Immunosorbent Assay for MBP-Specific Antibodies

The enzyme-linked immunosorbent assay was performed in 96-well plates. The plates were coated overnight with MBP (0.1 μg per well in 50 μL) in carbonate buffer (pH 8). The plates were then blocked with 1% BSA for 1 h at 37 °C and washed 3 times with PBS-T. Rat plasma (diluted 1:5 in PBS-T) was added and incubated for 1 h at 37 °C. The plates were washed again 3 times with PBS-T, and anti-rat peroxidase-conjugated antibodies (1:4000, PBS-T) were added for 1 h at 37 °C. Wells were then washed 5 times with PBS-T, tetramethylbenzidine (TMB) substrate was added, and the reaction was stopped with 10% orthophosphoric acid. Optical density was measured using a CLARIOstar Plus multimodal reader at 450 nm (BMG LABTECH GmbH, Ortenberg, Germany). All samples were analyzed in triplicate, and the average OD450 value was used for analysis.

### 2.7. Histological Studies

Rat spinal cord samples in a bone frame, corresponding in length to 5 vertebrae (access vertebra—Th13, 2 adjacent cranial vertebrae (Th11–12) and 2 adjacent caudal vertebrae (L1–2)), were fixed in 10% neutral formalin solution for 3–5 days, rinsed in running water, and subjected to subsequent decalcification in Trilon B at room temperature for 12–16 days. After satisfactory decalcification, the samples were cut out to remove soft tissues and orient the biomaterial for subsequent microtomy in the sagittal plane. The cut fragments were washed in running tap water, dehydrated in ethyl alcohols of increasing concentration, and embedded in homogenized paraffin embedding medium Bio Plast plus (Bio-Optica Milano S.p.A., Milan, Italy). Microtomy was performed using a rotary microtome with a Microm section transfer system HM 340E (Microm International GmbH, Walldorf, Germany) in the sagittal plane of the spinal cord, serially with a step of 100 μm. Paraffin sections for general microscopy were stained with hematoxylin and eosin; histochemical studies to detail the formation and maturation of the gliomesodermal scar were performed using Heidenhain’s Azan trichrome staining; and for a detailed assessment of the astrocytic glia in the cryodestruction focus, immunohistochemical detection of glial fibrillary acidic protein (GFAP) was used as a marker of astrocytic glia. To detect GFAP-positive cells, rabbit antibodies against rat were used, and the secondary antibodies were FITC-labeled goat antibodies. Stained preparations were placed in the synthetic mounting medium Bio Mount (Bio-Optica Milano S.p.A., Milan, Italy). Histological preparations were studied using conventional light microscopy with an AxioScope A1 microscope (Carl Zeiss Microscopy GmbH, Jena, Germany). Microphotographs were obtained using a high-resolution Axiocam 305 color camera (Carl Zeiss Microscopy GmbH, Jena, Germany), and morphometric measurements were performed using ZEN 2.6 lite software (Carl Zeiss Microscopy GmbH, Jena, Germany). For morphological measurements, a section in the sagittal plane with the largest defect area and 2 adjacent sections were selected—“above” and “below” the section with the largest defect area. Histological assessment was performed with the investigator blinded to the treatment group, and animals were randomly allocated to experimental groups. Morphometric methods included counting the number of mononuclear infiltrating cells per unit area of the glial scar, namely on microphotographs with an area of 37,500 μm^2^ (40× objective, 10× eyepiece) in at least 30 fields of view. On immunohistochemical preparations, the depth of penetration of GFAP-positive cells into the scar tissue was measured, and the number of GFAP-positive cells per unit area of the glial scar (500 × 500 μm) at the border of the defect with intact tissue was counted. In addition, the specific volume of vessels and collagen fibers in the structure of the glial scar was measured in at least 10 random fields of view using ZEN 2.6 lite software (Carl Zeiss Microscopy GmbH, Jena, Germany) as well as the ImageJ v.1.53e image processing program.

### 2.8. Cloning of Human and Rat CCR4 2–39 Thioredoxin Fusion Constructs

The N-terminal extracellular epitope regions of the human and rat CCR4 receptors (hCCR4 and rCCR4, residues 2–39) were assembled by overlap extension PCR using two primer pairs per construct (Table S1). Amplification was performed as follows: initial denaturation at 94 °C for 3 min; 25 cycles of 94 °C for 30 s, 67 °C for 30 s and 72 °C for 30 s; followed by 25 cycles of 94 °C for 30 s, 65 °C for 30 s and 72 °C for 30 s. The resulting fragments were digested with *BglII* and *XhoI* and ligated into pET-32b(+) (Novagen, Madison, WI, USA) in frame with the N-terminal thioredoxin (Trx) and His_6_ tag. All constructs were verified by Sanger sequencing.

### 2.9. Expression and Purification of Trx–CCR4_2–39_ Fusion Proteins

pET-32b(+) constructs coding for Trx-His_6_-hCCR4_2–39_, Trx-His_6_-rCCR4_2–39,_ or Trx-His_6_ fused with an irrelevant peptide (Supplementary Figure S1) were transformed into One Shot BL21 Star (DE3) *Escherichia coli* cells (Thermo Fisher Scientific, Waltham, MA, USA). Cells were grown in 500 mL of LB medium supplemented with 50 µg/mL ampicillin and 0.1% glucose at 37 °C and 200 rpm until OD_600_ = 0.6. Expression was induced with 1 mM isopropyl β-D-1-thiogalactopyranoside (IPTG) and continued for 4 h at 37 °C. Cells were harvested by centrifugation (4000× *g*, 15 min), resuspended in 20 mM Tris-HCl (pH 8.0), and lysed by sonication. Soluble and insoluble fractions were separated by centrifugation at 30,000× *g* for 10 min, and the soluble fraction was analyzed by SDS-PAGE with Coomassie Brilliant Blue staining.

Trx-fused peptides, carrying a hexahistidine tag, were purified by immobilized metal affinity chromatography (IMAC) on Ni^2+^-NTA Sepharose (GE Healthcare, Uppsala, Sweden) pre-equilibrated with 20 mM Tris-HCl (pH 8.0) containing 10 mM imidazole (Sigma–Aldrich, St. Louis, MO, USA). After washing with equilibration buffer, the bound protein was eluted with stepwise increasing imidazole concentrations (0.1, 0.3, and 0.5 M). Peak fractions were resolved by 15% SDS-PAGE (Coomassie Brilliant Blue), pooled, and dialyzed overnight against 20 mM Tris-HCl (pH 7.5), containing 150 mM NaCl.

### 2.10. CCR4-Binding ELISA

96-well plates were coated overnight at 4 °C with Trx-His_6_-hCCR4_2–39_, Trx-His_6_-rCCR4_2–39,_ or Trx-His_6_-irrelevant peptide, each at 0.1 µg per well in 50 µL of carbonate buffer (pH 9.0). Plates were blocked with 1% BSA (1 h, 37 °C) and washed three times with PBS-T. Mogamulizumab was applied as eleven threefold serial dilutions (50–0.0008 µg/mL) in PBS-T and incubated for 1 h at 37 °C. After three washes with PBS-T, bound antibody was detected with anti-human IgG (Fc-specific)–peroxidase antibody (A0170, Sigma–Aldrich, St. Louis, MO, USA; 1:60,000 in PBS-T; 1 h, 37 °C). Wells were washed five times with PBS-T, developed with tetramethylbenzidine (TMB) substrate, and the reaction was stopped with 10% orthophosphoric acid. Absorbance was read at 450 nm on a CLARIOstar Plus multimodal reader (BMG LABTECH GmbH, Ortenberg, Germany). Each concentration was assayed in triplicate, and four-parameter logistic curves were fitted in GraphPad Prism version 10.4.1 (GraphPad Software, Boston, MA, USA) to estimate EC_50_ values.

### 2.11. Anti-Human Antibody ELISA

Plasma concentrations of human IgG in DA rats i.v. administered 4 mg/kg of mogamulizumab or human IgG1κ isotype control (MedChemExpress, Monmouth Junction, NJ, USA) were determined by enzyme-linked immunosorbent assay (ELISA) using 96-well plates. The plates were coated overnight with anti-human IgG antibodies (I1886-2ML, Sigma–Aldrich, St. Louis, MO, USA) at a concentration of 1 μg/mL in carbonate buffer (pH 9.0), using a final volume of 100 μL per well. The plates were subsequently blocked with 1% bovine serum albumin (BSA) for 1 h at 37 °C and washed three times with phosphate-buffered saline containing Tween 20 (PBS-T).

Plasma samples collected before antibody administration (0) and on days 1, 3, and 8 from mogamulizumab-treated and isotype control-treated rats were initially diluted 1:100 in conjugation buffer, followed by serial threefold dilutions. For construction of the calibration curve, mogamulizumab was serially diluted fivefold in conjugation buffer to generate seven calibration concentrations starting from 0.5 μg/mL. Diluted plasma samples and mogamulizumab standards were incubated for 1 h at 37 °C. The plates were then washed three times with PBS-T and incubated with horseradish peroxidase (HRP)-conjugated anti-human IgG Fc-specific antibodies (A0170, Sigma–Aldrich, St. Louis, MO, USA; 1:10,000 in PBS-T) for 1 h at 37 °C. Following five washes with PBS-T, tetramethylbenzidine (TMB) substrate was added, and the reaction was stopped by the addition of 10% orthophosphoric acid. Optical density was measured at 450 nm using a CLARIOstar Plus multimode microplate reader (BMG LABTECH GmbH, Ortenberg, Germany). Plasma antibody concentrations were calculated using the mogamulizumab calibration curve and corrected for the corresponding plasma dilution factors. Concentrations measured in plasma samples from isotype control-treated animals were expressed as mogamulizumab-equivalent concentrations.

### 2.12. Isolation of Peripheral Blood Leukocytes and Mogamulizumab-Based Detection of CCR4 by Immunoblotting

Whole blood samples (4 mL) were collected in EDTA from mogamulizumab-treated and human IgG1κ isotype control-treated rats on day 8. Samples were centrifuged at 300× *g* for 3 min, after which the plasma fraction was removed. The remaining cellular fraction was resuspended in erythrocyte lysis buffer containing 150 mM NH_4_Cl, 10 mM KHCO_3_, and 0.1 mM EDTA, gently mixed, and incubated for 10 min at room temperature. Samples were subsequently centrifuged at 400× *g* for 5 min, and the supernatant was discarded. The erythrocyte lysis procedure was repeated three times until a macroscopically white leukocyte pellet without visible erythrocyte contamination was obtained. The resulting peripheral blood leukocyte pellets were resuspended in PBS-T and lysed by sonication. Protein concentrations were determined using the Bradford assay. The lysates were adjusted to equal protein concentrations, mixed with 4× sample buffer without β-mercaptoethanol (β-ME), and subjected to electrophoresis on 10% SDS-polyacrylamide gels. Following SDS-PAGE, proteins were transferred onto nitrocellulose membranes (Bio-Rad, Hercules, CA, USA) using a Trans-Blot Turbo semi-dry transfer system (Bio-Rad, Hercules, CA, USA). Following transfer, the membranes were blocked with 5% BSA in PBS-T for 1 h at room temperature. The membranes were then washed with PBS-T and incubated for 1 h in conjugation buffer containing 0.5% BSA and anti-human IgG antibodies (I1886-2ML, Sigma–Aldrich, St. Louis, MO, USA). After washing with PBS-T, one membrane was incubated overnight with mogamulizumab (20 μg/mL in PBS-T), whereas the corresponding control membrane was processed in parallel without mogamulizumab incubation. Both membranes were subsequently washed with PBS-T and incubated for 1 h with HRP-conjugated anti-human IgG Fc-specific antibodies (A0170, Sigma–Aldrich, St. Louis, MO, USA; 1:10,000). Following washing with PBS-T, chemiluminescent signals were developed using Clarity Western ECL substrate (Bio-Rad, Hercules, CA, USA) and visualized by chemiluminescence using a ChemiDoc Imaging System (Bio-Rad, Hercules, CA, USA). Additionally, the same samples were analyzed by Western blotting using an HRP-conjugated anti-β-actin antibody (A3854, Sigma–Aldrich, St. Louis, MO, USA; 1:10,000) as a loading control.

### 2.13. Statistics

Statistical analysis was performed with SPSS Statistics 28 software (IBM, Armonk, NY, USA). Tukey’s honestly significant difference test (Tukey’s HSD) was used to assess differences between treated and non-treated animals.

## 3. Results

### 3.1. Immunoproteasome Is Elevated in the Injured Spinal Cord During Acute and Subacute Stages

After spinal cord injury, a pronounced neuroinflammatory response develops, characterized by neutrophil infiltration into the injury zone, activation of microglia and astrocytes, and massive secretion of proinflammatory cytokines and production of reactive oxygen species. All these processes lead to the accumulation of damaged proteins, requiring effective proteolytic elimination to prevent increased cellular stress and secondary neuronal death. In this context, the immunoproteasome plays an important role, not only maintaining intracellular proteostasis by degrading damaged proteins [13], but also participating in the regulation of immune cell differentiation and modulating proinflammatory signaling cascades. To gain a deeper understanding of the pathophysiological processes accompanying traumatic CNS injury, we performed a comprehensive analysis of the dynamics of the concentrations of the main proinflammatory cytokines in the injured area and their temporal relationship with catalytic and regulatory subunits of the immunoproteasome in the spinal cord tissues of DA and SD rats subjected to spinal cord cryoinjury performed through surgical access at the Th13 vertebral level.

**Figure 1:**
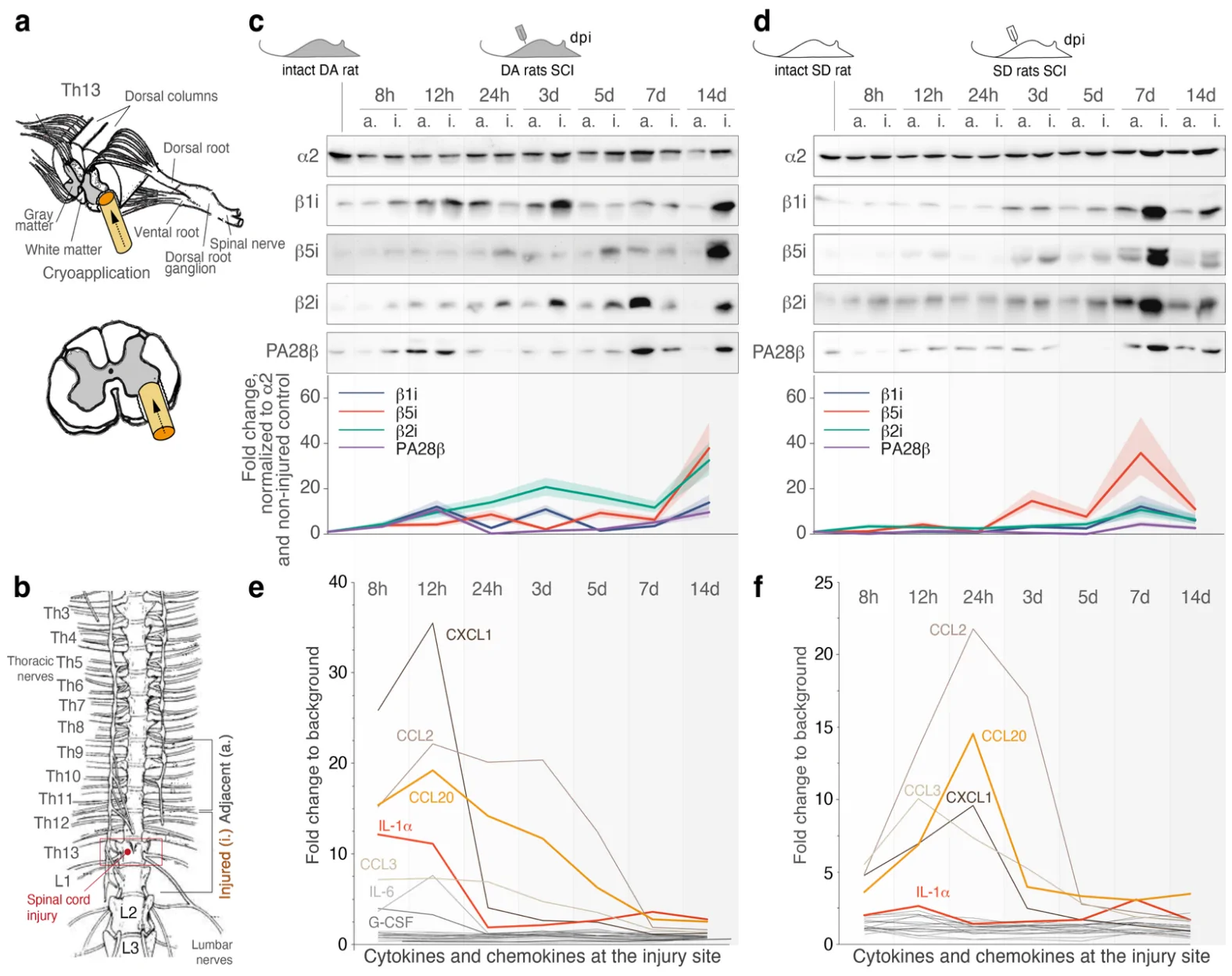
Induction of immunoproteasome in spinal cord after SCI is synchronized with IL-1α release. (**a**) Three-dimensional model of laminectomy and cryoapplication of the Th13 vertebra. (**b**) Schematic representation of injured and adjacent areas of the rat spine. (**c**,**d**) Immunoblotting for proteasome subunits α2, β1i, β5i, β2i, and PA28β of SD and DA rats in the injured (i.) and adjacent (a.) spinal cord 8 h–14 days post SCI. Bottom panels represent fold-change levels in the injured spinal cord normalized to the α2 subunit and non-injured control. Colored zones represent standard deviation from two technical replicates. (**e**,**f**) Intensity of release (fold change to background) of IL-1α (red), CXCL-1, CCL2, CCL20 (yellow), CCL3, IL-6, and G-CSF in injured spinal cord in SD and DA rats after SCI. Other measured cytokines and chemokines (thin black lines; please refer to the Materials and Methods Section 2.4 for details) did not increase significantly.

We used the previously developed experimental model of SCI in rodents, in which the spinal cord of animals is damaged by a copper cryoapplication probe supercooled with liquid nitrogen, introduced through a drilled slot in the spine using a microsurgical bur with a diamond coating [14] (Figure 1a). We analyzed spinal cord tissue samples (the directly injured Th12-L1 region and the adjacent Th9-Th11 region) obtained at different time intervals after injury (8, 12, 24 h, 3, 5, 7, and 14 days). A multiplex method was used for quantitative analysis of cytokine and chemokine concentrations in the injured area, and the levels of immunoproteasome subunits were determined for both the injured site and the adjacent site (Figure 1b).

Immunoproteasome assembly involves the replacement of constitutive catalytic subunits (β1, β2, and β5) with inducible ones (β1i/LMP2, β2i/MECL-1, and β5i/LMP7). Analysis of the expression of immunoproteasome subunits by immunoblotting (Figure 1c,d and Supplementary Figure S2) revealed that the catalytic immunosubunits began to be noticeably expressed by 24 h after injury, with expression levels at the injury site gradually increasing and reaching a peak on days 7 and 14 in SD and DA rats, respectively. We observed several strain-specific patterns in the induction of the immunoproteasome. In DA rats, we detected a significant increase in β1i and β2i expression in the injured area on day three, and β1i was already upregulated 12 h post-SCI. By day seven, β2i expression was also elevated in the adjacent segment of the injured spinal cord. In contrast, SD rats exhibited expression of all three immunosubunits in the injured area only on the seventh day post-SCI, while no other remarkable peaks in immunoproteasome expression were detected in the adjacent or damaged segments throughout the study period. Expression of the immunoproteasome-associated regulatory subunit PA28β generally followed the dynamics of the catalytic immunosubunits. Notably, similar to β1i, it was also upregulated during the acute phase of SCI (12 h) in DA rats. Overall, these findings indicate that immunoproteasome induction in DA rats occurs more rapidly and consistently than in SD rats.

The inflammatory response after neurotrauma, in particular spinal cord injury, is characterized by a complex and interrelated activation of various cytokines and chemokines. We next aimed to correlate immunoproteasome upregulation with data on the elevation of pro-inflammatory cytokines and chemokines (Figure 1e,f and Supplementary Figure S3). The concentrations of the chemokines CXCL1/GROα, CCL2/MCP-1 and CCL20/MIP-3α in the injured area in DA rats reached their maximum (~32-fold, 20-fold, and 18-fold increase, respectively) after 12 h, while in SD rats CXCL1 (~10-fold increase), CCL2 and CCL20 (22- and 15-fold increases, respectively) reached peak values 24 h post-injury. Notably, the concentration of all measured cytokines decreases in SD by days three to five. In contrast, in DA rats, the concentrations of these chemokines decreased more slowly, only by day seven, except for CXCL1 and IL-1α—both dropped sharply by 24 h after injury. The level of CCL3/MIP-1α in the injured spinal cord in both strains reached approximately the same concentration and showed similar dynamics. A moderate increase in the concentrations of IL-1α, IL-6, and G-CSF was also observed in DA rats during the first hours after injury, with a gradual decrease to the baseline level by 24 h. It is interesting to note that IL-1α and CCL20, but not IFNγ or other cytokines, showed a secondary release burst during the subacute phase of SCI. In both rat strains, these inflammatory mediators were still detectable by day seven and correlated with immunoproteasome upregulation.

### 3.2. Administration of Mogamulizumab Suppresses Induction of Anti-Myelin Antibodies After SCI

Traumatic SCI is believed to increase the risk of MS [15]: both T cells and B cells recognizing one of the key MS autoantigens, myelin basic protein (MBP), are elevated in mice [16] and patients [17] with SCI even 10 years after the initial injury [18]. On the other hand, SCI [19], similarly to MS [20], may be treated by induction of immunological tolerance toward MBP. Recently, we demonstrated that ubiquitin-independent [21] charge-mediated [22] hydrolysis of MBP by PA28αβ-capped immunoproteasomes and its further presentation significantly contribute to the pathogenesis of autoimmune neurodegeneration [23]. Linking these data with the current results, we further propose that anti-chemokine receptor therapy limiting immune cell infiltration into the central nervous system may be an effective strategy for SCI treatment, not only by restricting chemotaxis to the injured area, but also by suppressing the myelin-specific autoreactive immune response. We thus investigated whether mogamulizumab, a humanized defucosylated monoclonal antibody directed against C-C motif chemokine receptor 4 (CCR4)—a major chemokine receptor expressed by T helper type 2 (Th2) polarized cells [24]—could counteract the induction of anti-myelin antibodies after SCI in DA rats.

**Figure 2:**
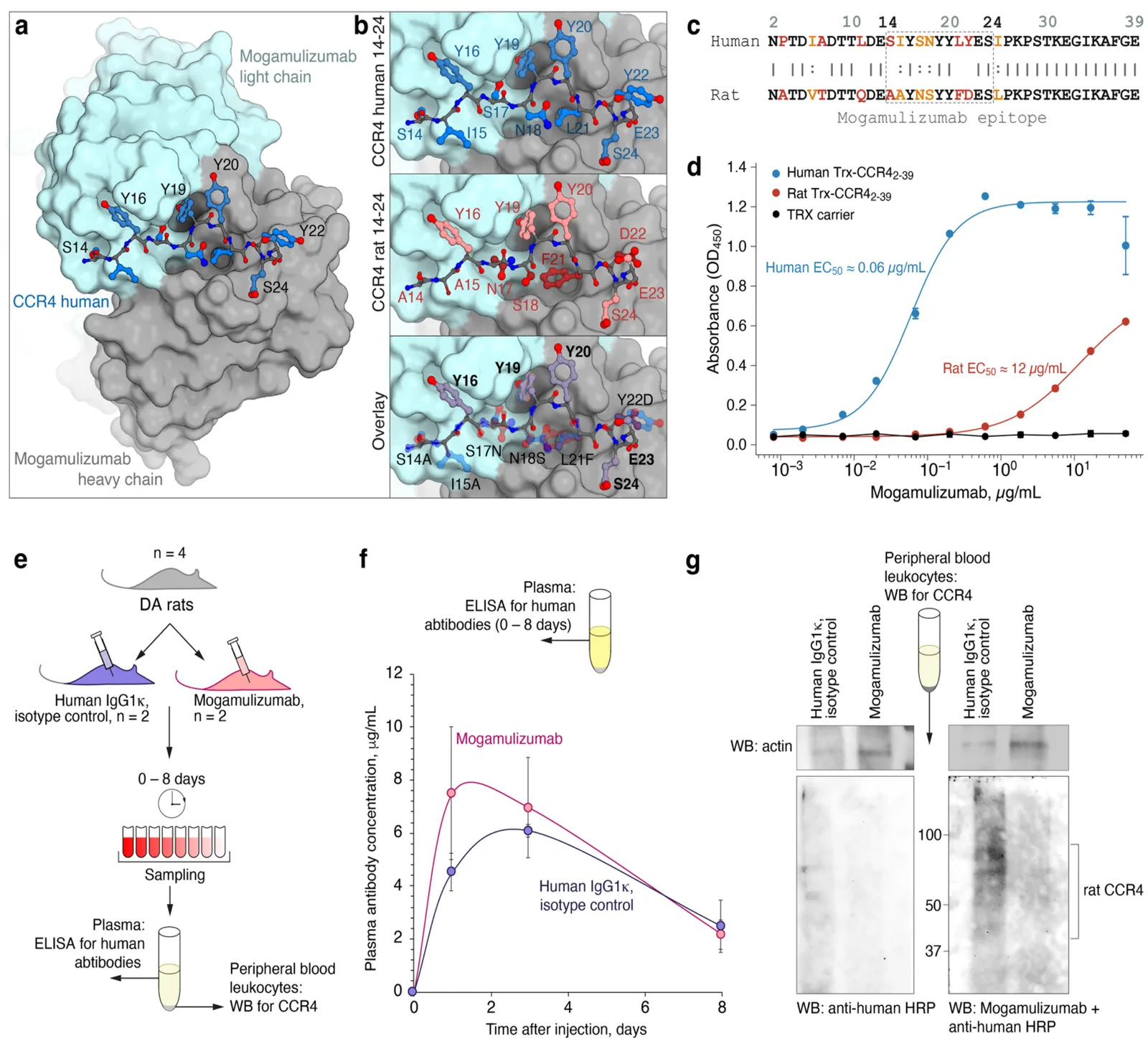
Mogamulizumab cross-reacts with rat CCR4 through a conserved epitope core and affects CCR4-positive peripheral blood leukocytes in vivo. (**a**) Crystal structure of the mogamulizumab Fab in complex with the human CCR4 N-terminal peptide 14-24 (PDB: 9ULM). (**b**) Close-up of the CCR4 epitope 14–24 for human (**top**, blue), rat (direct substitution, middle, red), and their overlay (**bottom**). Conserved contact residues are highlighted in bold. (**c**) Pairwise alignment of the human and rat CCR4 N-terminal extracellular region (residues 2–39). The mogamulizumab epitope is indicated. (**d**) ELISA binding of a threefold serial dilution of mogamulizumab (50–0.0008 µg/mL) to plate-immobilized Trx-fused human CCR4_2–39_ (blue), rat CCR4_2–39_ (red), and the Trx carrier with an irrelevant peptide (control, black). Data are mean ± SD of triplicates; apparent EC_50_ values are indicated. (**e**) Experimental design of plasma antibody kinetics and CCR4-associated immunoreactivity in rat peripheral blood leukocytes. DA rats received mogamulizumab or a human IgG1κ isotype control (*n* = 2 animals per group). Blood samples were collected over an 8-day observation period. Plasma antibody concentrations were measured by anti-human IgG ELISA, and peripheral blood leukocytes collected on day 8 were analyzed by immunoblotting. (**f**) Plasma antibody concentrations (average and standard deviation, samples in triplicate) following administration of mogamulizumab or the isotype control. Values for the human IgG1κ isotype control are expressed as mogamulizumab-equivalent concentrations. (**g**) Representative immunoblots of peripheral blood leukocyte samples collected 8 days after antibody administration. Detection was performed using HRP-conjugated anti-human IgG alone (**left**; secondary-antibody-only control) or mogamulizumab (**right**), followed by HRP-conjugated anti-human IgG.

To determine if mogamulizumab engages the rat orthologue of its target, we compared its binding to the human and rat CCR4 N-terminal extracellular region (residues 2–39), which contains the mogamulizumab epitope. We analyzed the structure of the mogamulizumab Fab in complex with the human CCR4_14–24_ peptide (Figure 2a) and placed the rat orthologue onto the same paratope (Figure 2b). Within the epitope, the paratope-contacting residues Y16, Y19 and Y20 were identical in both species, whereas six contact positions differed. The human and rat peptides share 71% identity and 84% similarity, with all substitutions confined to the membrane distal region (positions 2–24) and a fully conserved C-terminal segment (Figure 2c).

We next measured binding directly by ELISA against recombinant human and rat CCR4_2–39_ fused to a thioredoxin (Trx) carrier, using the Trx carrier alone as a control (Figure 2d). Mogamulizumab bound both orthologues in a specific, dose-dependent manner, with no signal against the Trx carrier. Binding to human CCR4 was saturable with an apparent EC_50_ of ≈ 0.06 µg/mL. Binding to rat CCR4 was also dose-dependent and significantly above control across the informative range (0.6–50 µg/mL) but did not reach saturation. Its apparent EC_50_ (≈12 µg/mL) is therefore a lower-bound estimate and indicates substantially weaker binding to the rat CCR4 fragment than to the human one.

Human IgG1 antibodies administered to rats show a small volume of distribution close to the plasma/extracellular volume (~70–110 mL/kg) and long terminal half-lives of ~7–14 days. Indeed, DA rats administered mogamulizumab or a human IgG1κ isotype control at 4 mg/kg (Figure 2e) demonstrated the highest sampled antibody concentration on day one, at approximately 7.5 μg/mL, followed by a decline to approximately 2.2 μg/mL on day eight (Figure 2f). The isotype control profile reached its highest sampled value on day three, at approximately 6.0 μg/mL, and decreased to approximately 2.5 μg/mL by day eight. Thus, both administered antibodies remained at half of mogamulizumab EC_50_ for at least three days. Immunoblotting of day eight peripheral blood leukocyte samples revealed a weaker CCR4-associated signal in the mogamulizumab-treated sample than in the isotype control sample. The secondary-antibody-only control did not reproduce the prominent immunoreactivity observed following probing with mogamulizumab (Figure 2g).

**Figure 3:**
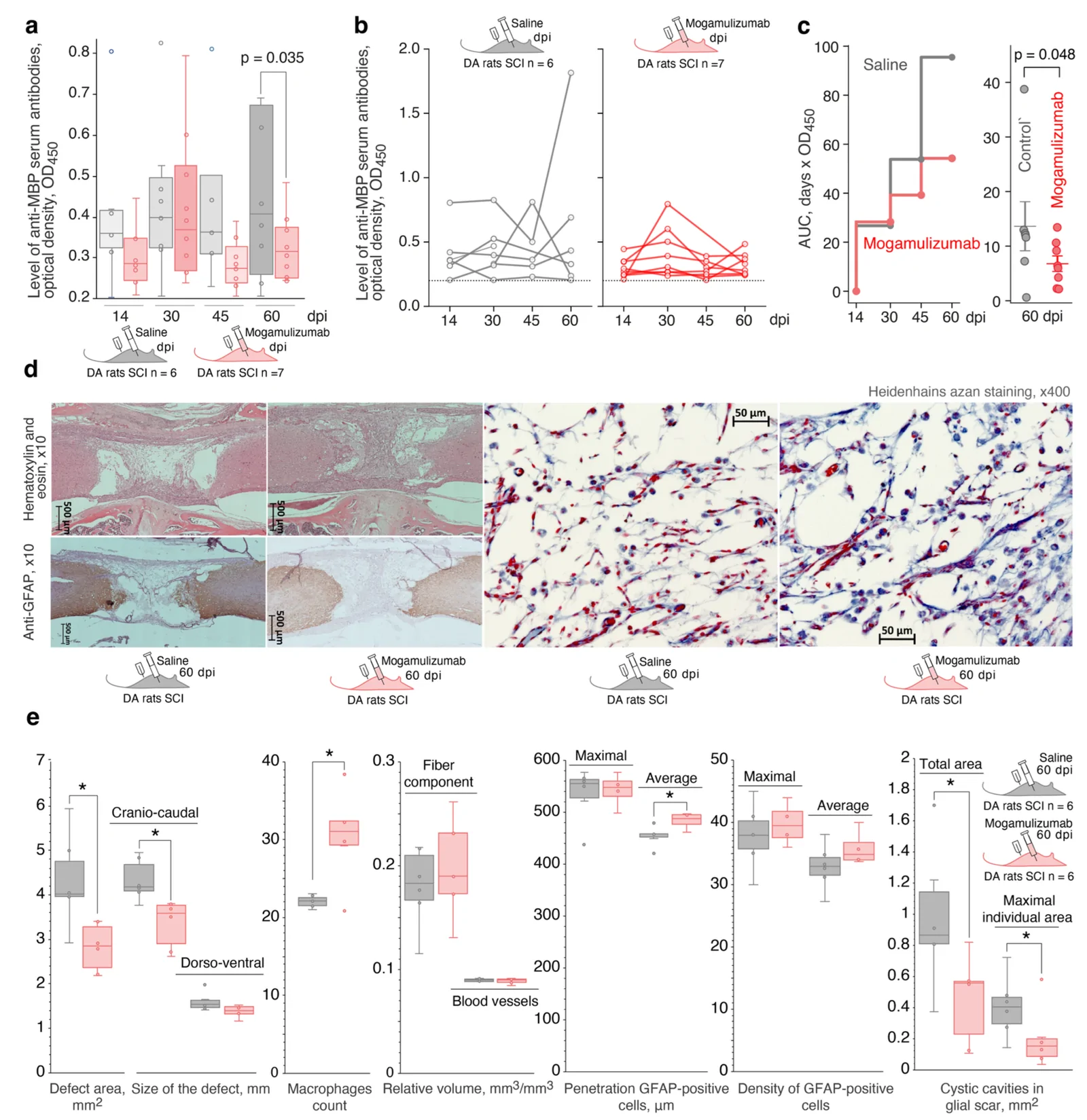
SCI treatment with mogamulizumab decreases the level of antibodies to myelin basic protein and reduces defect area and cyst formation in DA rats. (**a**,**b**) Levels of plasma anti-MBP antibodies after SCI in rats treated with mogamulizumab (red) and animals receiving saline (gray). Each dot represents the average for an individual animal, measured in triplicate. The background level (no plasma sample) is indicated (dashed line). (**c**) Cumulative (**left**) and individual (**right**) area under the curve (AUC) data. Values on the right are shown for 60 dpi with mean ± SEM. Statistical significance was assessed using a one-sided permutation test. (**d**) Images of spinal cord defects in rats, stained with hematoxylin and eosin, according to Heidenhain with azan, and for GFAP-positive cells as indicated. (**e**) Histological examination of spinal cord defect area in rats treated with mogamulizumab in comparison with non-treated animals. dpi—days post-injury. Bars represent median, interquartile range, and data spread. The asterisk denotes a statistically significant difference according to Tukey’s honestly significant difference (Tukey’s HSD) test.

We then experimentally treated DA rats with SCI. Animals were injected intravenously twice with mogamulizumab, immediately after SCI and five days after injury, at the same dose of 4 mg/kg. Plasma samples for evaluation of anti-MBP antibodies were collected at 14–60 days after cryoinjury. The results of the enzyme-linked immunosorbent assay showed that plasma samples from rats with SCI treated with mogamulizumab were characterized by lower levels of antibodies to MBP compared with those from the control group (Figure 3a,b), and by day 60 of the experiment, this discrepancy reached statistical significance (Figure 3c).

The therapeutic potential of mogamulizumab was evaluated by pathohistological analysis 60 days post-SCI, when the glial scar at the injury site was completely formed. For morphometric analysis of the Th13 vertebra at the level of cryoinjury, one site in the sagittal plane characterized by the maximum defect area and one adjacent site located above the main injured region were selected. Analysis of samples from injured animals revealed increased collagen fiber density, large cystic cavities, and a mature fibrous component compared with samples from rats treated with mogamulizumab (Figure 3d). Selective immunohistochemical staining of astrocytic glia in the injury area for glial fibrillary acidic protein (GFAP) demonstrated that in treated rats it was less damaged than in untreated animals (Figure 3d). These data confirm a potential role for mogamulizumab [4] in improving astroglial plasticity and reducing inflammation after injury.

The structure of spinal cord defects in treated and non-treated rats was heterogeneous. The average area of the glial scar decreased by 1.5 times in rats treated with mogamulizumab compared with animals receiving saline (2.8 ± 0.5 versus 4.3 ± 1.0 mm^2^), and the cranio-caudal size of the defect was 0.9 mm smaller (3.4 ± 0.6 versus 4.3 ± 0.5 mm) (Figure 3d, Table 1). In the formed glial scar, as a rule, numerous racemose cavities of variable size were found, localized mainly at the border of the defect with intact tissue in the projection of the dorsal funiculi of the white matter in both the cranial and caudal directions. Small racemose cavities were in some cases located in the projection of the ventral funiculi of the white matter at the border with intact tissue. Administration of mogamulizumab restricted both the number of racemose cavities and their size (Figure 3d, Table 1).

The average total area of cyst-like cavities decreased by 2.1 times with mogamulizumab administration (0.46 ± 0.28 versus 0.97 ± 0.45 mm^2^), and the average area of the largest cyst in the defect structure decreased by two times compared with untreated animals (0.2 ± 0.2 versus 0.4 ± 0.2 mm^2^). Mononuclear infiltrating cells in the defect area were significantly elevated in treated rats and were unevenly distributed, with minor variation in morphology and size (Figure 3d, Table 1). Rats treated with mogamulizumab demonstrated minor positive dynamics in the depth of astroglia penetration into the defect structure and in the quantitative expression of the density of astrocytes at the border of the defect with intact tissue. GFAP-positive cells showed a trend toward greater penetration into the central part of the defect 484 ± 16 μm in mogamulizumab-treated animals versus 454 ± 19 μm in controls, but this difference did not reach statistical significance. Administration of mogamulizumab to rats with SCI did not affect the specific volume of blood vessels or the density and distribution of collagen fibers.

## 4. Discussion

Within 12–24 h of SCI, we observed a robust increase in proinflammatory mediators at the injury site, with CXCL1 showing the greatest divergence between strains: DA rats exhibited ~fourfold higher CXCL1 levels than SD rats. CXCL1 is rapidly produced by microglia, astrocytes, and endothelial cells after neurotrauma and chiefly signals through CXCR2 to recruit neutrophils [25]. In this context, higher CXCL1 may plausibly prime a more pronounced neutrophil infiltration in DA rats, amplifying secondary injury via barrier disruption and oxidative/excitotoxic pathways, as well as activating the NLRP3 inflammasome via the specific receptor CXCR2 [26]. Although the total levels of CCL2, CCL20, and CCL3 were comparable between DA and SD strains, their concentration dynamics differed. The CCL2-CCR2 axis recruits monocytes/macrophages and sustains inflammation; in turn, further activation of the NF-κB pathway in astrocytes and microglia may exacerbate demyelination [26,27]. Like CCL2, CCL20, interacting with the CCR6 receptor, induces migration of IL-17-producing T lymphocytes and contributes to the maintenance of chronic inflammation [28]. CCL3 participates in the acute phase of SCI and viral brain inflammation by recruiting neutrophils and promoting neurodegenerative processes, and its loss is associated with reduced infiltration and improved functional outcomes in rodents [29,30]. Our data suggest that CCL2, CCL20, and CCL3 returned to baseline within a week in DA rats, which was slower than in SD rats (three to five days). This suggests that DA rats not only experience a stronger early neutrophil-centric signal, driven by CXCL1, but also experience a more prolonged chemokine milieu that could influence subacute tissue responses. We also analyzed the dynamics of less pronounced mediators, IL-6 and G-CSF, at the site of injury, which were elevated only in DA rats 8–12 h post-SCI. IL-6 is a central cytokine regulating the inflammatory response and neurogenesis. Early elevation of IL-6 promotes activation of astrocytes and microglia, supporting both acute and subacute inflammatory responses and promoting immune cell infiltration across the blood–brain barrier, leading to demyelination and axonal injury [31].

In addition to assessing the overall dynamics of the local cytokine storm, we investigated whether these dynamics would correlate with the induction of the immunoproteasome. Interestingly, IL-1α, a proinflammatory mediator that activates microglia and reactive astrocytosis [32], was initially less pronounced than other mediators, but it was the only cytokine that started to increase by day seven post-injury. Despite the absence of a distinct IFN-γ peak at the injury site, classically the most potent inducer of immunoproteasomes [33], we observed that immunoproteasome induction followed IL-1α dynamics. Recently, it was shown that IL-1α can inhibit the Grin2c/Ca2+/CaMK2b signaling pathway, inducing astrocyte proliferation and scar formation, and IL-1α blockade may offer a new therapeutic strategy for improving the prognosis of spinal cord injury [34]. Importantly, activated microglia have been shown to induce neurotoxic reactive A1 astrocytes via IL-1α, TNF, and C1q in response to induced neuroinflammation [35]. Unlike reactive A2 astrocytes, which actively promote neuronal survival and tissue repair, A1 astrocytes secrete a neurotoxin that causes rapid neuronal and oligodendrocyte death [36]. Furthermore, it has recently been shown that IL-1α is sufficient to trigger a cascade of cellular and molecular events that lead to the rapid death (within 24 h) of nearly 40% of mature oligodendrocytes in the mouse spinal cord. In addition, IL-1α-stimulated astrocytes produce reactive oxygen species (ROS), causing astrocyte- and endothelial cell-mediated oligodendrocyte degeneration [32]. Presumably, immunoproteasome induction occurs in response to ROS production by reactive astrocytes [37], although the exact mechanism of activation remains unclear. Finally, induction of the immunoproteasome with an enriched β1i phenotype, synchronized with upregulation of PA28β as early as 12 h post-SCI in DA rats, may promote the generation of immunodominant MBP peptides due to ubiquitin-independent proteasomal hydrolysis in oligodendrocytes [23] and therefore may be considered a primary event of the myelin-reactive immune response.

Recently, we showed that after spinal cord injury in humans, a CCL22 chemokine gradient, established primarily by dendritic cells (DCs) and macrophages, is detectable in the CSF from the first week after trauma [4]. This gradient can recruit CCR4-expressing Th2 cells, which may be activated by myelin peptides presented on MHC class II molecules and further provide help to B cells, promoting their differentiation into plasma cells that secrete anti-myelin antibodies. CCR4 is expressed predominantly by a defined subset of memory/skin-homing T lymphocytes [38] and is the target of the defucosylated monoclonal antibody mogamulizumab. Its principal mode of action is depletion of CCR4-expressing (Th2- and Treg-polarized) cells through antibody-dependent cellular cytotoxicity (ADCC) [39].

The structural and binding data explain both why mogamulizumab retains activity against rat CCR4 and why its apparent affinity is reduced. The three aromatic residues that anchor the epitope in the paratope pocket (Y16, Y19, and Y20) are conserved between human and rat, whereas the divergent positions (S14A, I15A, S17N, N18S, L21F, Y22D) lie in the peripheral part of the epitope. The substitutions at the C-terminal edge of the NYYLYES motif are the most consequential, replacing a bulky aromatic and an aliphatic contact with a charged residue and a different residue, respectively (Y22D, L21F). In other words, the aromatic anchor holds while the surrounding contacts are weakened, which accounts for the ~100-fold drop in EC_50_ without loss of specific binding.

Several considerations argue that this reduced affinity is nonetheless sufficient for target engagement in the rat at the dose used. First, administration of mogamulizumab at 4 mg/kg provides post-distribution concentrations of 6–8 μg/mL in plasma for days, which are near the apparent EC_50_ measured for rat CCR4 (≈ 12 µg/mL). Second, Fc afucosylation may help sustain effector recruitment despite weaker antigen recognition. In a comparative cross-species study, afucosylated human IgG1 elicited greater FcγRIIIA/FcγRIV-dependent reporter activation than matched fucosylated antibodies, including through rat FcγRIV [40]. Furthermore, low-fucose anti-CCR4 IgG1 has been shown to mediate NK-cell-dependent ADCC at lower target-antigen densities in human effector cell assays [41]. These findings provide a mechanistic rationale for Fc-dependent activity of mogamulizumab in rats. However, whether enhanced Fc receptor engagement can compensate for weaker recognition of rat CCR4 remains to be established, particularly because antigen-binding affinity itself influences ADCC [42]. Third, membrane receptor organization could contribute to avidity-enhanced antibody binding. Studies of other chemokine receptors have demonstrated density-dependent, transient CXCR4 homodimerization [43], CCR5 self-association and CXCR4–CCR5 heteromeric complexes [44], and CXCR4 nanoclustering in resting T cells [45]. Although these observations do not establish analogous organization of rat CCR4, they provide a basis for considering an avidity-dependent mechanism. Provided that two CCR4 epitopes are accessible and appropriately spaced, bivalent engagement by mogamulizumab could stabilize cell–surface binding [46]. Such an effect could contribute to activity despite weaker apparent binding to the rat CCR4 fragment in ELISA.

**Figure 4:**
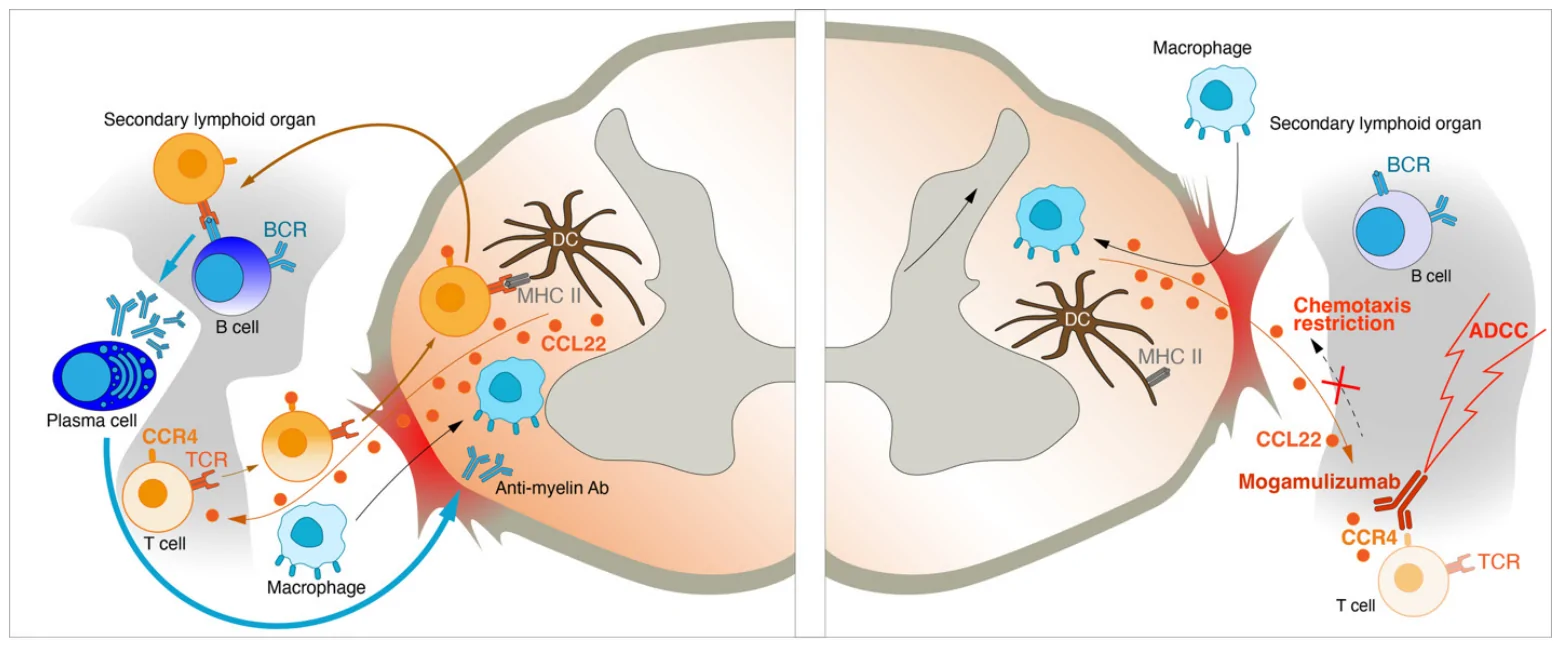
Hypothetical model linking CCR4-directed therapy to the SCI-associated humoral autoimmune response. After SCI, a CCL22 gradient established mainly by resident dendritic cells (DCs) and infiltrating macrophages recruits CCR4-expressing T cells, which are activated by MHC class II-presented myelin peptides. These autoreactive T cells can help B cells to differentiate into plasma cells producing anti-myelin antibodies. Mogamulizumab depletes CCR4+ (Th2/Treg) cells through ADCC, reducing the pool of T cells available for recruitment along the CCL22 gradient and thereby their infiltration into the injury site, which in turn suppresses the production of myelin-reactive antibodies. CCL17/CCL22 levels and CCR4+ cell infiltration were not directly measured in the present rat study. The depicted axis is inferred from similarity between cytokine and chemokine release into CSF during SCI in humans and rats [4].

The weaker CCR4-associated signal in the peripheral blood leukocytes from mogamulizumab-treated rats, demonstrated in this study, is consistent with an alteration in the circulating CCR4-expressing cell compartment. A reduced pool of circulating CCR4+ T cells could limit their recruitment along a CCL22 gradient into the injured spinal cord and thereby contribute to the numerically lower anti-MBP antibody reactivity observed in treated animals (Figure 4). Conversely, the increased abundance of mononuclear infiltrating cells within the lesion raises the possibility that mogamulizumab-mediated modulation of CCR4+ regulatory T cells altered the local inflammatory milieu. However, all these findings should be considered preliminary evidence of an association between antibody administration and reduced CCR4-positive peripheral blood leukocytes, rather than proof of a specific depletion mechanism mediated by mogamulizumab in rats.

The therapeutic effect of mogamulizumab was accompanied by the previously reported [4] remarkable modification of the glial scar histoarchitecture in DA rats compared with non-treated animals. Mogamulizumab administration resulted in a long-term excessive presence of mononuclear infiltrating cells within the glial scar, apparently reflected in a more pronounced attraction and differentiation of fibroblasts in the early period after cryotrauma, with subsequent activation of the synthesis and maturation of the fibrous component. This assumption is consistent with the minimization of the linear dimensions of the spinal cord defect and the size and number of cystic cavities in the structure of the glial scar in mogamulizumab-treated animals.

## 5. Conclusions

Our study suggests that, together with the overall beneficial therapeutic effect observed previously [4], treatment of SCI with mogamulizumab suppresses the autoimmune humoral myelin-reactive response, pointing to a complementary immunological modality for spinal cord protection after injury.

## Figures and Tables

**Table 1:** Histological examination of spinal cord defect area in rats treated with mogamulizumab in comparison with untreated animals. All data are provided as the mean ± standard deviation. Asterisks denote statistically significant differences.

Experimental Group	Saline	Mogamulizumab
Defect area, mm^2^	4 ± 1 (*n* = 6)	2.8 ± 0.5 (*n* = 6) *
Cranio-caudal defect size, mm	4.3 ± 0.5 (*n* = 6)	3.4 ± 0.6 (*n* = 6) *
Dorso-ventral defect size, mm	1.6 ± 0.2 (*n* = 6)	1.4 ± 0.1 (*n* = 6)
Maximum area of individual cavities, mm^2^	0.4 ± 0.2 (*n* = 6)	0.2 ± 0.2 (*n* = 6) *
Total area of racemose cavities in the glial scar, mm^2^	1.0 ± 0.4 (*n* = 6)	0.5 ± 0.3 (*n* = 6) *
Racemose cavities to total area of the glial scar, %	22 ± 5 (*n* = 6)	15 ± 8 (*n* = 6)
Mononuclear infiltrating cells per 37,500 µm^2^ of defect area	22.1 ± 0.8 (*n* = 7)	31 ± 6 (*n* = 6) *
Fibrous component volume, mm^3^/mm^3^	0.18 ± 0.04 (*n* = 6)	0.20 ± 0.05 (*n* = 5)
Blood vessel volume, mm^3^/mm^3^	0.090 ± 0.001 (*n* = 6)	0.089 ± 0.003 (*n* = 5)
Average penetration depth of GFAP-positive cells, µm	454 ± 19 (*n* = 6)	484 ± 16 (*n* = 4)
Maximum penetration depth of GFAP-positive cells, µm	535 ± 52 (*n* = 6)	542 ± 33 (*n* = 4)
Average density of GFAP-positive cells	33 ± 4 (*n* = 6)	36 ± 3 (*n* = 4)
Maximum density of GFAP-positive cells	38 ± 5 (*n* = 6)	40 ± 4 (*n* = 4)

## Data Availability

All data relevant to the study are included in the article.

## Supplementary Materials

The following supporting information can be downloaded at: https://www.mdpi.com/article/10.3390/cells15211999/s1, Supplementary Figure S1: Annotated DNA and protein sequences of the Trx–His_6_ fusion antigens; Supplementary Figure S2: Time-dependent dynamics of proteasome subunits following experimental spinal cord injury; Supplementary Figure S3: Absolute cytokine concentrations, normalization procedures, sample size, comparison between groups, and correlation analyses; Supplementary Table S1: Primers used for cloning of the hCCR4 and rCCR4 epitope constructs used in this study.

## Abbreviations

The following abbreviations are used in this manuscript:

SCI	spinal cord injury
MS	multiple sclerosis
MBP	myelin basic protein
CSF	cerebrospinal fluid
BSCB	Blood–spinal cord barrier
CNS	central nervous system
GFAP	glial fibrillary acidic protein
ELISA	enzyme-linked immunosorbent assay
ADCC	antibody-dependent cellular cytotoxicity
CCR4	C-C motif chemokine receptor 4
DA	Dark Agouti
SD	Sprague-Dawley
ROS	reactive oxygen species
DC	dendritic cell
Th2	T helper type 2
Treg	regulatory T cell
