# Supplementary material for: Mogamulizumab Suppresses Induction of Myelin-Reactive Antibodies After Experimental Spinal Cord Injury
Source: Cells. 2026 Oct 31;15(21):1999. doi: 10.3390/cells15211999 (PMC13761874; doi:10.3390/cells15211999)
Supplement: Supplementary file 1 [file cells-15-01999-s001.zip › cells-4454598-supplementary.pdf]

Mogamulizumab Suppresses Induction of Myelin-Reactive Antibodies after Experimental Spinal Cord Injury

by Darya A. Orlova, Aleksandr S. Chernov, Vitaly A. Kazakov, Georgii B. Telegin, Alexey A. Belogurov Jr., and Anna A. Kudriaeva

Supplementary Materials

Supplementary Figures S1-S3

Supplementary Table S1

Supplementary Figure S1. Annotated DNA and protein sequences of the Trx–His₆ fusion antigens used in this study

Key

- T7 promoter
- lac operator
- RBS (Shine–Dalgarno)
- thioredoxin A
- His₆ tag
- thrombin site
- S-tag
- linker / cloning scar
- antigen insert
- mogamulizumab epitope (CCR4 14–24)
- stop codons + XhoI

(A) Trx–His₆–hCCR4$_{2–39}$ 667 bp / 189 aa

DNA

```
  1  TAATACGACTCACTATAGGGGAATTGTGAGCGGATAACAATTCCCCTCTAGAAATAATTT   60
 61  TGTTTAACTTTAAGAAGGAGATATACATATGAGCGATAAAATTATTCACCTGACTGACGA  120
121  CAGTTTTGACACGGATGTACTCAAAGCGGACGGGGCGATCCTCGTCGATTTCTGGGCAGA  180
181  GTGGTGCGGTCCGTGCAAAATGATCGCCCCGATTCTGGATGAAATCGCTGACGAATATCA  240
241  GGGCAAACTGACCGTTGCAAAACTGAACATCGATCAAAACCCTGGCACTGCGCCGAAATA  300
301  TGGCATCCGTGGTATCCCGACTCTGCTGCTGTTCAAAAACGGTGAAGTGGCGGCAACCAA  360
361  AGTGGGTGCACTGTCTAAAGGTCAGTTGAAAGAGTTCCTCGACGCTAACCTGGCCGGTTC  420
421  TGGTTCTGGCCATATGCACCATCATCATCATCATTCTTCTGGTCTGGTGCCACGCGGTTC  480
481  TGGTATGAAAGAAACCGCTGCTGCTAAATTCGAACGCCAGCACATGGACAGCCCAGATCT  540
541  GAACCCGACCGACATCGCTGATACCACTCTGGACGAGAGCATCTACTCCAACTATTACCT  600
601  GTATGAATCTATTCCGAAACCGTCTACTAAAGAAGGCATCAAAGCGTTCGGTGAATAATG  660
661  ACTCGAG    667
```

Protein

```
  1  MSDKIIHLTDDSFDTDVLKADGAILVDFWAEWCGPCKMIAPILDEIADEYQGKLTVAKLN   60
 61  IDQNPGTAPKYGIRGIPTLLLFKNGEVAATKVGALSKGQLKEFLDANLAGSGSGHMHHHH  120
121  HHSSGLVPRGSGMKETAAAKFERQHMDSPDLNPTDIADTTLDESIYSNYYLYESIPKPST  180
181  KEGIKAFGE    189
```

(B) Trx–His₆–rCCR4$_{2–39}$ 667 bp / 189 aa

DNA

```
  1  TAATACGACTCACTATAGGGGAATTGTGAGCGGATAACAATTCCCCTCTAGAAATAATTT   60
 61  TGTTTAACTTTAAGAAGGAGATATACATATGAGCGATAAAATTATTCACCTGACTGACGA  120
121  CAGTTTTGACACGGATGTACTCAAAGCGGACGGGGCGATCCTCGTCGATTTCTGGGCAGA  180
181  GTGGTGCGGTCCGTGCAAAATGATCGCCCCGATTCTGGATGAAATCGCTGACGAATATCA  240
241  GGGCAAACTGACCGTTGCAAAACTGAACATCGATCAAAACCCTGGCACTGCGCCGAAATA  300
301  TGGCATCCGTGGTATCCCGACTCTGCTGCTGTTCAAAAACGGTGAAGTGGCGGCAACCAA  360
361  AGTGGGTGCACTGTCTAAAGGTCAGTTGAAAGAGTTCCTCGACGCTAACCTGGCCGGTTC  420
421  TGGTTCTGGCCATATGCACCATCATCATCATCATTCTTCTGGTCTGGTGCCACGCGGTTC  480
481  TGGTATGAAAGAAACCGCTGCTGCTAAATTCGAACGCCAGCACATGGACAGCCCAGATCT  540
541  GAACGCTACCGACGTTACTGACACCACTCAGGACGAAGCTGCTTACAACTCTTACTACTT  600
601  CGATGAATCTCTGCCGAAACCGTCTACCAAAGAAGGTATCAAAGCATTCGGTGAATAATG  660
661  ACTCGAG    667
```

Protein

```
  1  MSDKIIHLTDDSFDTDVLKADGAILVDFWAEWCGPCKMIAPILDEIADEYQGKLTVAKLN   60
 61  IDQNPGTAPKYGIRGIPTLLLFKNGEVAATKVGALSKGQLKEFLDANLAGSGSGHMHHHH  120
121  HHSSGLVPRGSGMKETAAAKFERQHMDSPDLNATDVTDTTQDEAAYNSYYFDESLPKPST  180
181  KEGIKAFGE    189
```

(C) Trx–His₆–irrelevant peptide 592 bp / 164 aa

DNA

```
  1  TAATACGACTCACTATAGGGGAATTGTGAGCGGATAACAATTCCCCTCTAGAAATAATTT   60
 61  TGTTTAACTTTAAGAAGGAGATATACATATGAGCGATAAAATTATTCACCTGACTGACGA  120
121  CAGTTTTGACACGGATGTACTCAAAGCGGACGGGGCGATCCTCGTCGATTTCTGGGCAGA  180
181  GTGGTGCGGTCCGTGCAAAATGATCGCCCCGATTCTGGATGAAATCGCTGACGAATATCA  240
241  GGGCAAACTGACCGTTGCAAAACTGAACATCGATCAAAACCCTGGCACTGCGCCGAAATA  300
301  TGGCATCCGTGGTATCCCGACTCTGCTGCTGTTCAAAAACGGTGAAGTGGCGGCAACCAA  360
361  AGTGGGTGCACTGTCTAAAGGTCAGTTGAAAGAGTTCCTCGACGCTAACCTGGCCGGTTC  420
421  TGGTTCTGGCCATATGCACCATCATCATCATCATTCTTCTGGTCTGGTGCCACGCGGTTC  480
481  TGGTATGAAAGAAACCGCTGCTGCTAAATTCGAACGCCAGCACATGGACAGCCCAGATCT  540
541  GGGCTTCGAGATCGACAAGGTGTGGTACGACCTGGACGCCTAATGACTCGAG    592
```

Protein

```
  1  MSDKIIHLTDDSFDTDVLKADGAILVDFWAEWCGPCKMIAPILDEIADEYQGKLTVAKLN   60
 61  IDQNPGTAPKYGIRGIPTLLLFKNGEVAATKVGALSKGQLKEFLDANLAGSGSGHMHHHH  120
121  HHSSGLVPRGSGMKETAAAKFERQHMDSPDLGFEIDKVWYDLDA    164
```

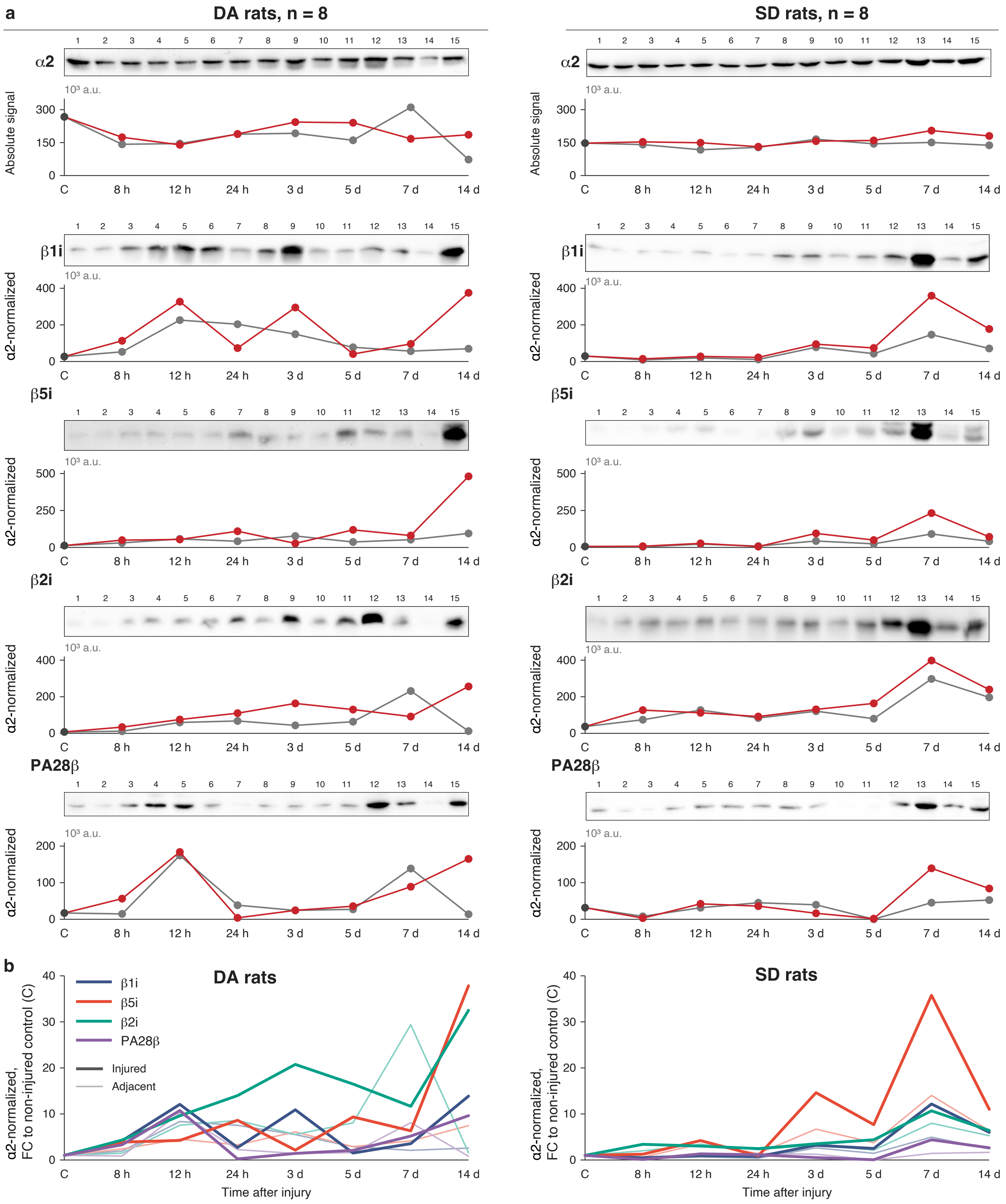

Supplementary Figure S2. Time-dependent dynamics of proteasome subunits following experimental spinal cord injury. (a) Immunoblots and densitometric analysis of α2, β1i, β5i, β2i and PA28β in injury site and adjacent region in DA (left column, n = 7) and SD (right column, n = 7) rats. Lane 1 represents sample from intact control (n = 1/1, DA/SD). Lanes 2–15 contain consecutive pairs of tissue adjacent to the injury site (adjacent, 2/4/6/8/10/12/14) and tissue from the injury site (injured, 3/5/7/9/11/13/15), collected at 8, 12 and 24 h and 3, 5, 7 and 14 d after injury. The graphs beneath the α2 blots show absolute background-corrected integrated band signals in 10^3 arbitrary units, normalized to α2 level. Adjacent tissue is shown in grey and injured tissue in red. (b) Combined profiles of β1i, β5i, β2i and PA28β in DA and SD rats (fold change, FC, average from 2 technical replicates) following a second normalization to non-injured control. Blue, coral, green and purple lines represent β1i, β5i, β2i and PA28β, respectively. Adjacent tissue is shown by thin line.

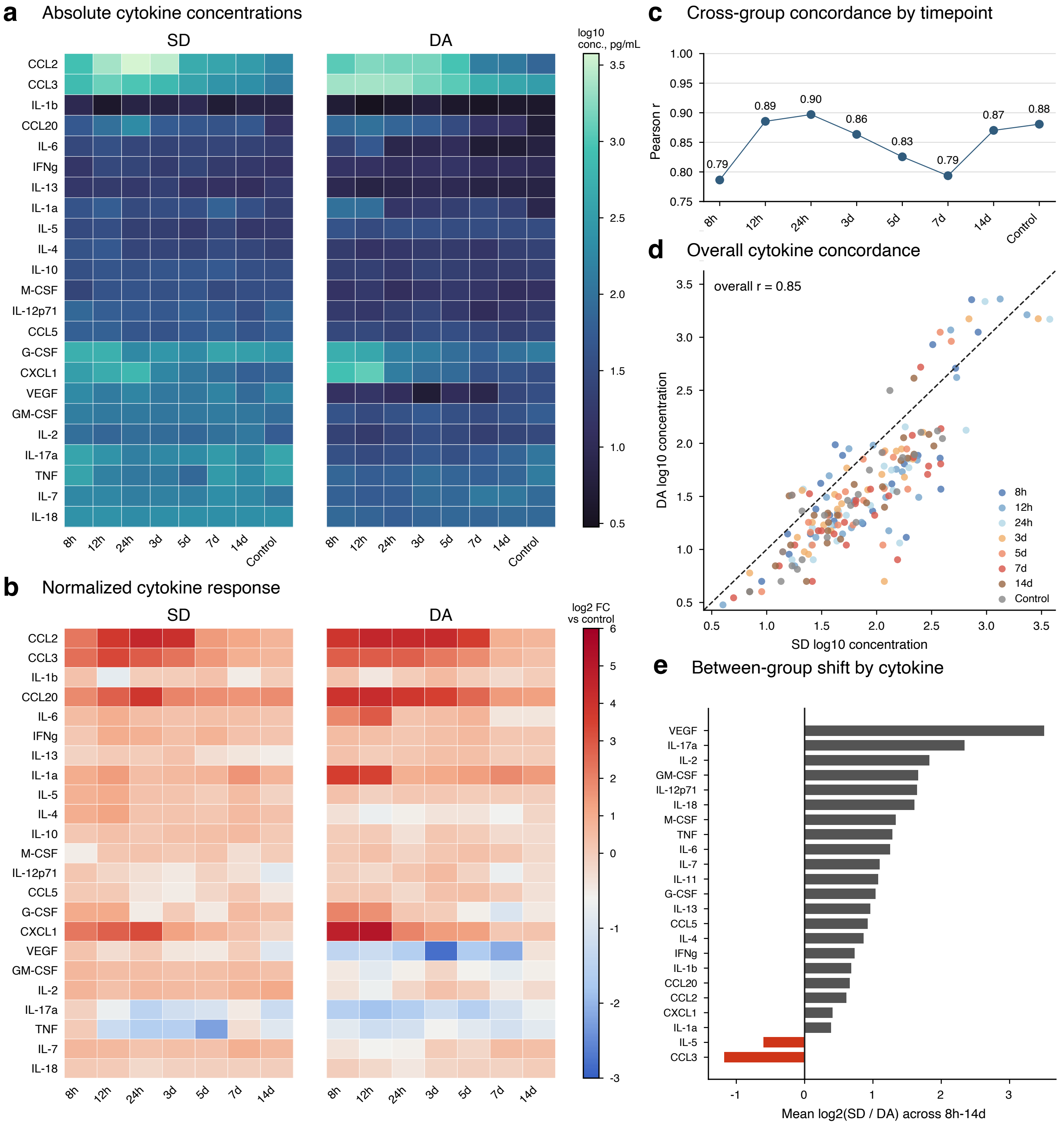

Supplementary Figure S3. Absolute cytokine concentrations, normalization procedures, sample size, comparison between groups, and correlation analyses. (a) Absolute cytokine and chemokine concentrations in spinal cord extracts (injured site, Th12-L1) from Sprague-Dawley (SD) and Dark Agouti (DA) rats at 8 h, 12 h, 24 h, 3 days, 5 days, 7 days, and 14 days after spinal cord injury (SCI), together with the corresponding uninjured controls. All 23 analytes measured using the multiplex cytokine assay are shown (3 technical replicates). (b) Cytokine responses normalized independently within each rat strain. For each analyte, values at individual post-injury time points were divided by the corresponding strain-specific Control without SCI value and expressed as $\log_2$ fold change. (c) Descriptive concordance between the SD and DA cytokine profiles at each sampling time. Pearson correlation coefficients were calculated across the complete set of 23 analytes after $\log_{10}$ transformation of the absolute concentration values. Concordance remained high throughout the observation period, with Pearson r values ranging from approximately 0.79 to 0.90. (d) Overall relationship between cytokine concentrations in SD and DA rats. Each point represents one cytokine at one sampling time, plotted as the $\log_{10}$ concentration in SD rats against the corresponding value in DA rats. Points are colored according to sampling time and the dashed diagonal denotes the line of identity. The overall descriptive Pearson correlation across all cytokine-time pairs was r = 0.85. (e) Cytokine-specific differences between rat strains. For each analyte, the mean $\log_2$ ratio of the SD concentration to the DA concentration was calculated across all post-injury time points from 8 h to 14 days. Positive values indicate higher average concentrations in SD rats, whereas negative values indicate those for DA rats.

Supplementary Table S1. Primers used for cloning of the hCCR4 and rCCR4 epitope constructs

**Primer**	**Sequence**
hCCR4_BglII_fr1	5′-AATTAGATCTGAACCCGACCGACATCGCTGATACCACTCTGGACGAGAGC-3′
hCCR4_XhoI_rev1	5′-AATTCTCGAGTCATTATTCACCGAACGCTTTGATGCCTTCTTTAGTAGAC-3′
hCCR4_fr2	5′-CTGATACCACTCTGGACGAGAGCATCTACTCCAACTATTACCTGTATGAATCTATTCCG-3′
hCCR4_rev2	5′-CGCTTTGATGCCTTCTTTAGTAGACGGTTTCGGAATAGATTCATACAGGTAATAGTTGGAG-3′
rCCR4_BglII_fr1	5′-AATTAGATCTGAACGCTACCGACGTTACTGACACCACTCAGGACGAAGC-3′
rCCR4_XhoI_rev1	5′-AATTCTCGAGTCATTATTCACCGAATGCTTTGATACCTTCTTTGGTAGAC-3′
rCCR4_fr2	5′-CTGACACCACTCAGGACGAAGCTGCTTACAACTCTTACTACTTCGATGAATCTCTGCCG-3′
rCCR4_rev2	5′-CGAATGCTTTGATACCTTCTTTGGTAGACGGTTTCGGCAGAGATTCATCGAAGTAGTAAGAGTTG-3′
